# Incidence of Vitamin D Deficiency in Different Seasons in the Adult Karachi Population Presenting in the Medical Outpatient Department with Generalized Body Ache

**DOI:** 10.7759/cureus.5167

**Published:** 2019-07-18

**Authors:** Asif Raza, Jabbar Ghufran Syed, Farah Muhammad Ali, Muhammad Danish Khan, M Ali Khan, Farhan Haleem, Rubiya Naeem

**Affiliations:** 1 Internal Medicine, Jinnah Postgraduate Medical Centre, Karachi, PAK; 2 General Surgery, Jinnah Postgraduate Medical Centre, Karachi, PAK; 3 Internal Medicine, Civil Hospital Karachi, Dow University of Health Sciences, Karachi, PAK; 4 Orthopaedics, The Indus Hospital, Karachi, PAK; 5 Gastroenterology, Jinnah Postgraduate Medical Centre, Karachi, PAK; 6 Cardiology, Jinnah Postgraduate Medical Centre, Karachi, PAK

**Keywords:** vitamin d, seasons, karachi, adult, generalized body ache, variation, winter, summer

## Abstract

Introduction

The most important function of vitamin D is to maintain normal calcium homeostasis. Various factors play an important role but the most significant aspect of its normal physiological functioning is exposure to sunlight, therefore, it is also known as the sunshine vitamin. In adults, a prolonged deficiency of vitamin D (calcitriol) can lead to osteomalacia while a lower deficiency (insufficiency) is associated with various non-specific symptoms.

Vitamin D deficiency has been observed in developed and developing countries, including the Middle East and the subcontinent. Vitamin D is mandatory for the maintenance of health due to the presence of its highly specific receptors, Vitamin D receptors, in all body tissues and its regulatory role in the encoding of more than 200 genes. The deficiency of Vitamin D, therefore, could affect any tissue or body system. Most interventions for this are done through outpatient departments (OPDs).

The burden of vitamin D deficiency is affected by seasonal variation in our part of the world as well as internationally; data show a marked variation, however. Generalized body ache is a vague symptom. It is one of the most common complaints seen at the OPD and can be a manifestation of many a disease. But a correlation with low vitamin D levels has been observed previously. Whether this relation is affected by seasonal variation remains unascertained and data on the above-mentioned relationship for Pakistan are scarce.

Objective

We aim to evaluate the incidence of vitamin D deficiency in different seasons in the adult Karachi population presenting in medical OPDs with a generalized body ache.

Materials and methods

This study was conducted at Medical Ward 5, Jinnah Postgraduate Medical Center, Karachi, from January 2016 to December 2016. Data were collected from the OPD that was held twice-weekly (Mondays and Fridays). Only patients who exclusively complained of "generalized body ache" were inducted into the study. Patients with minor complaints, such as headache, backache, fatigue, and lethargy, were also seen only if there were no comorbidity at all. Meticulous lab and clinical workup were done to rule out potentially not-so-benign causes of the symptoms.

Patients 18 years or older were inducted into the study. Once written consent was taken, Vitamin D levels were carried out via the COBAS (Roche Diagnostics, Mannheim, Germany) method. A vitamin D level of ≤30 ng/ml was considered deficient. Results were obtained within a week, and data were recorded and analyzed. Summer was defined as three months either side of the summer solstice (June 21) and winter was defined as three months either side of the winter solstice (December 21).

Results

A total of 577 patients were inducted into the study. The mean age of the patients was 39.33 ± 10.23 years. The patients were predominantly female (72.7%) and housewives. Of these, 298 (51.64%) had a vitamin D deficiency; in summer, the incidence was 44.23% and in winter, it was 60.37%. The mean level of vitamin D in deficient patients was 25.06±8.74 ng/dl.

Conclusion

Vitamin D levels are significantly decreased in patients complaining of generalized body ache even without any comorbidity. These affect predominantly the middle-aged female population. Seasonal variation occurs with most patients presenting during the winter months, along with lower means.

## Introduction

Vitamin D is known as the sunshine vitamin. Through the exposure of skin to sunlight/sunshine (UV light), cholecalciferol or vitamin D3 is produced, which can then be converted to Calcitriol. This then acts via nuclear receptors to exert physiological and anatomical effects. Adequate exposure to sunlight produces all the requirements of the body. Because of this fact, it is not considered an essential part of a regular diet.

It is also a fat-soluble vitamin. Dietary sources include egg yolk, oily fish, butter, and milk [1]. Its most important function is to maintain the normal calcium balance in the body [2]. Vitamin D is mandatory for the maintenance of health due to the presence of its highly specific receptors, vitamin D receptors (VDRs), in all body tissues and the regulatory role it plays in the encoding of more than 200 genes. The deficiency of vitamin D, therefore, could affect any tissue or body system [3].

In adults, a prolonged deficiency of vitamin D (Calcitriol) can lead to osteomalacia [4] while a lower deficiency (insufficiency) is associated with various non-specific symptoms [5], including generalized body ache, fatigue, and lethargy. As such, vague symptoms may mask pathological levels. The correlation of symptom severity with vitamin D levels is uncommon, however.

Vitamin D deficiency has been identified as a global problem, with an estimated one billion people worldwide suffering from it. It has also been observed in developed and developing countries, including the subcontinent and the Middle East [6]. Avoidance of sunshine or inadequate intake of vitamin D and malnutrition are the main causes in these regions.

A micronutrient so dependent on sunlight is bound to have seasonal variation [7]. Regions with higher temperatures and longer days tend to have paradoxically low levels of vitamin D due to the avoidance of heat and sunlight by the local population. Pakistan is one such country. No extensive research or study has been done on the seasonal variation of vitamin D; there is more than enough data on the prevalence, regional distribution, age groups, and risk factors though.

## Materials and methods

Consent was taken in writing by all patients. All data were kept confidential. All labs and workup were carried out free of cost to the patients. This study was approved by the ethical board committee of Jinnah Postgraduate Medical Centre.

Study design

Cross-sectional

Setting

Research was conducted in the outpatient department (OPD) of Medical Unit 5 of Jinnah Postgraduate Medical Centre. OPDs were held twice weekly on Mondays and Fridays.

Seasons and duration

This study was held for 12 months from January 2016 to December 2016. Two seasons were defined, summer and winter. Both seasons were defined with respect to the hours of sunlight available in the day rather than the temperature variations seen throughout the year.

Summer was defined as three months on either side of the summer solstice (June 21). Winter was defined as three months on either side of the winter solstice (December 21). On average, the hours of sunlight available in summer was 15.5±1.75 hours, and for winter, it was 12.5±1.75 hours.

Sampling technique

Non-probability, consecutive sampling

Inclusion criteria

Patients of either gender, presenting with generalized body ache, aged 18 years or older, were enrolled in the study.

Generalized Body Ache

It is one of the most common complaints seen at OPDs, however, there is no absolute definition for it. It is often used vaguely by patients and even doctors, in some instances, to define mild fatigue, lethargy, or dull aches. Body ache, in general, can have multiple causes.

Here, we use the term to define pain involving multiple joints or body parts or even the entire body (as described by the patients) that is chronic (three months or more) in nature, dull, not localized, does not cause any debilitation to normal function, does not radiate, cannot be elicited, nor is associated with bone tenderness. It must be defined at a scale of three or less on the visual analog scale (VAS) of pain.

The pain or ache must have been such that it would only require over-the-counter painkillers. Any pain or ache requiring hospital admission, intravenous access, antipsychotics, antidepressants, or other medications would not be eligible by our definition. The pain must not be attributable to any comorbidity (see exclusion criteria).

Exclusion criteria

1. Previously diagnosed cases of Vitamin D deficiency or insufficiency.

2. Patients with a history of orthopedic trauma or who had undergone major orthopedic procedures.

3. Patients with significant comorbidities such as diabetes, hypertension, cardiovascular disease, pulmonary disorders, and systemic disorders (multiple myeloma, scleroderma, and so on).

4. Patients with rheumatologic disorders, including osteoarthritis.

5. Patients who had taken corticosteroids at within the last five years for any medical reasons.

6. Drug addicts, psychiatric patients, alcoholics, and smokers.

7. Patients with significant gastrointestinal disorders such as celiac disease, ulcerative colitis, and so on.

8. Patients with neurological disease such as motor neuron disease, Parkinson’s, and so on.

9. Patients with any malignancy irrespective of stage and occurrence.

Vitamin D deficiency

The predominant form of vitamin D metabolites (25-hydroxyvitamin D or 25(OH)D) are a good measure of the overall vitamin D levels in the body. This was measured in our labs. Vitamin D levels were done using COBAS (Roche Diagnostics, Risch-Rotkreuz, Switzerland). A level of 30 ng/dl or less was considered deficient.

While most assays in the west use a level of 20 ng/dl as deficient, all labs in Karachi have raised this limit to 30 ng/dl based on multiple studies and experience. Both insufficiency and deficiency will be covered by levels of less than 30 ng/dl.

Ruling out comorbidity

Where clinical suspicion was strong and history suggested further tests, complete blood count, bone scan, thyroid profile, scan, radiological tests, blood cultures, and viral markers were done. A psychiatric consult was taken in certain patients. Patients with any significant comorbidities were excluded.

## Results

A total of 557 patients were enrolled into the study according to the induction criteria. A slightly higher incidence of “Generalized Body Ache” was seen in the summer months. A large chunk of the patients was female, subsequently, housewives were the most predominant occupation. Residences were almost equally split. Demographic characteristics and seasonal presentation are given in Table 1.

**Table 1:** Demographic characteristics

	N=577
Age (mean)	39.33±10.23 years
Gender	
Male	157 (27.3%)
Female	420 (72.7%)
Season (at presentation)	
Summer	312 (54.08%)
Winter	265 (46.92%)
Residence	
Urban	309 (53.5%)
Rural	268 (46.5%)
Occupation	
Housewives	288 (49.91%)
Students	149 (25.82%)
Unemployed	79 (13.69%)
Shopkeepers/Self-employed	53 (9.18%)
Others	08 (1.38%)

Overall, the incidence of Vitamin D deficiency was 51.64% and the mean level of vitamin D was 25.06±8.74 ng/ml. Seasonal variation and vitamin D levels are summarized in Table 2.

**Table 2:** Incidence of vitamin D deficiency with respect to seasonal variation and mean levels

Summer (N=312)	
Patients with Vitamin D deficiency	138 (44.23%)
Mean level of Vitamin D in deficient patients	29.74±10.65 ng/ml
Winter (N=265)	
Patients with Vitamin D deficiency	160 (60.37%)
Mean level of Vitamin D in deficient patients	23.45±7.9 ng/ml
Total (N=577)	
Patients with Vitamin D deficiency	298 (51.64%)
Mean level of Vitamin D in deficient patients	25.06±8.74 ng/ml

The incidence was disproportionately high for females (and housewives) throughout the seasons. Females also displayed lower vitamin D levels. Gender analyses are summarized in Table 3 and occupational analyses are summarized in Table 4.

**Table 3:** Seasonal variation of vitamin D levels with respect to gender

	N(%)
Summer (N=138)	
Male	35 (25.36%)
Female	103 (74.64%)
Winter (N=160)	
Male	31 (19.37%)
Female	129 (80.63%)
Overall (N=298)	
Male	66 (22.14%)
Female	232 (77.86%)

**Table 4:** Incidence of vitamin D deficiency in various occupations

N=298	N (%)	Occupational Incidence (%)
Housewives	171 (57.38%)	59.37 %
Students	84 (28.18%)	56.37 %
Unemployed	32 (10.73%)	40.50 %
Shopkeepers/ Self-employed	11 (3.69%)	20.75 %
Others	Nil	Nil

## Discussion

Vitamin D deficiency is seen far too often in OPDs and clinics throughout the world; it is even more pronounced in Pakistan [8]. As reported by Jaddon et al., it affects both genders, irrespective of age. However, in our study, females were disproportionately affected. Females were inducted at a ratio of almost 3:1. Patients were usually younger than what has been reported previously but this was statistically not significant.

With a median of 450 plus patients presenting at each OPD, overall more than 40,000 presented in the year this study was conducted. The inclusion criteria (see above) made sure that the most benign and “healthy” of patients were inducted only. Five-hundred and seventy-seven (577) is less than 2% of the patients seen at the OPD; it is not representative of the actual vitamin D deficiency faced by our population.

Having said that, it is worth seeing that body ache is usually self-treated by most patients who don’t have comorbidities. It was alarming to find that such a symptom carried a high underlying vitamin D pathology. There were no significant differences between the patients inducted during summer or winter. Similarly, no significant difference was associated with the urban or the rural population. These two factors can simply be attributed to the exceptionally high number of patients presenting at the OPD of the largest tertiary care center in Sindh.

Variation was seen in both seasons. In winter, not only was the incidence increased but the mean vitamin D levels were lower as well. One would expect this with fewer hours of sunlight, resulting in lower vitamin D levels, especially when correlated with the occupations. Certain jobs, or a lack thereof, are associated with fewer hours of exposure to sunlight. An improvement in vitamin D levels is seen from winter to summer [9], and this also explains the lower incidence and higher means seen during summertime.

Although the seasonal variation is significant, looking at the bigger picture, one should not take winter or summer into account when screening for vitamin D deficiency. With over half the screened participants exhibiting pathological levels, such a high prevalence mounts to an endemic even without comorbidities or symptomology. As previously described, ≤2% of all OPD patients were screened; one could only imagine the serious implications if wider and more extensive screening were done.

Housewives were the most affected occupation; even with students and unemployed patients, there was a female predominance. Within the occupation (housewives and students) themselves, the incidence was higher than the general population. The reasons for this are quite obvious in our society. We will abstain from social commentary, and simply advice that inadequate exposure to sunlight be ameliorated.

This study does not delve deep into the vitamin D problem. We simply analyzed the incidence of this endemic with respect to the benign symptom of “generalized body ache.” To our surprise, this general complaint masked a dangerous culprit, especially in the female population. But nearly half the patients did not have vitamin D deficiency and no clear pathology could be ascertained in these patients.

As to why only half the patients had vitamin D deficiency and the rest did not, no clear-cut answers came forth. Vitamin D levels also did not correlate with the symptoms. Therefore, we could not establish causality between generalized body ache and vitamin D levels. Further studies are warranted.

We assume that vitamin D deficiency and generalized body ache occur independently of each other. This does not mean that practicing physicians forego screening for vitamin D deficiency in these patients, as more than half exhibits low levels of vitamin D, which can be a therapeutic target.

Shortcomings

The authors would like to acknowledge the following shortcomings in the study:

1. Neither the nutritional status of the patients was assessed, nor food history.

2. Menopausal history was not analyzed.

## Conclusions

Vitamin D deficiency has a high incidence in patients with generalized body ache even in the absence of comorbidities. Lower incidence and better levels are seen in summer as compared to winter. Seasonal variation happens, but it should not determine the decision to screen or not. Although body ache occurs independently of vitamin D levels, further studies are required to precisely establish a correlation.
